# Databases for intrinsically disordered proteins

**DOI:** 10.1107/S2059798321012109

**Published:** 2022-01-21

**Authors:** Damiano Piovesan, Alexander Miguel Monzon, Federica Quaglia, Silvio C. E. Tosatto

**Affiliations:** a Department of Biomedical Sciences, University of Padova, Padova, Italy; b Institute of Biomembranes, Bioenergetics and Molecular Biotechnologies, National Research Council (CNR–IBIOM), Bari, Italy

**Keywords:** intrinsically disordered proteins, databases, protein ensembles, flexible proteins

## Abstract

Intrinsically disordered regions (IDRs) lacking a fixed three-dimensional protein structure are widespread and play a central role in cell regulation. Only a small fraction of IDRs have been functionally characterized, with heterogeneous experimental evidence that is largely buried in the literature. Predictions of IDRs are still difficult to estimate and are poorly characterized. Here, an overview of the publicly available knowledge about IDRs is reported, including manually curated resources, deposition databases and prediction repositories. The types, scopes and availability of the various resources are analyzed, and their complementarity and overlap are highlighted. The volume of information included and the relevance to the field of structural biology are compared.

## Abstract 2

The types, scopes, availability and complementarity of databases for intrinsically disordered proteins are described.

## 1. Introduction

Intrinsically disordered proteins (IDPs) and regions (IDRs) lack a fixed three-dimensional structure (Dyson & Wright, 2005 ▸). They dynamically sample a wide ensemble of conformations, forming local transient secondary structures (Dyson & Wright, 2005 ▸; Davey, 2019 ▸). IDRs are widespread across species, play a central role in cell regulation and are subject to extensive pre- and post-translational modifications (Van Roey *et al.*, 2012 ▸; Weatheritt & Gibson, 2012 ▸; Csizmok & Forman-Kay, 2018 ▸). IDRs are commonly involved in transient interactions underlying signal transduction processes (Wright & Dyson, 2015 ▸; Schad *et al.*, 2018 ▸; Davey, 2019 ▸) and provide unique structural attributes that form flexible linkers or fly-casting regions to capture binding partners (Shoemaker *et al.*, 2000 ▸). Recently, important roles of IDRs in mediating liquid–liquid phase separation (LLPS) and in contributing to the formation of membraneless organelles have been discovered (Boeynaems *et al.*, 2018 ▸; Borcherds *et al.*, 2021 ▸). The unstructured nature of IDRs, together with their ability to sample a broad range of conformations, allow them to interact with other IDRs, ordered proteins or nucleic acids through different multivalent interactions (Schuster *et al.*, 2020 ▸).

Despite their importance, only a small fraction of IDRs have been functionally characterized (Davey, 2019 ▸; Kumar *et al.*, 2020 ▸) and the available knowledge is largely buried in the literature. The different levels of quality and coverage of IDPs are a consequence of the heterogeneity of experimental fields studying protein structure and function (Felli & Pierattelli, 2015 ▸; Plitzko *et al.*, 2017 ▸). The structural aspects of IDRs are studied using a number of different biophysical methods, including nuclear magnetic resonance (NMR; Felli & Pierattelli, 2015 ▸), small-angle X-ray scattering (SAXS; Bernadó & Svergun, 2011 ▸), circular dichroism (CD; Ezerski *et al.*, 2020 ▸) and Förster resonance energy transfer (FRET; Holmstrom *et al.*, 2018 ▸).

Biological databases play a central role in accelerating biological discovery, making experimental information accessible in a standardized and structured way (Baxevanis, 2011 ▸). Databases provide sustained access to material resources, facilitating their reuse, and are essential for re-analysis, validation and testing of new hypotheses. There are two types of biological databases. Repositories, archives or deposition databases collect primary (*i.e.* experimental) data. Knowledge bases instead aggregate, process and visualize the primary data. Data in repositories normally remain static, whereas knowledge bases are dynamic and information is interpreted (often through manual curation) to create added value. Sometimes resources are of both types, and the majority of deposition databases also process data to facilitate visualization. Expert biocurators play a crucial role in IDP databases by providing a direct interpretation of disorder derived from structural experiments and manually curating these ID annotations. As an example, the missing electron densities derived from X-ray crystallographic experiments are interpreted as conformational heterogeneity in the crystal lattice, but do not provide a direct quantitative measure of structural dynamicity.

Knowledge on IDPs is scattered across different specialized databases that focus on different, often subtle, functional/structural aspects (or flavors). However, the lack of a standard classification, a clear nomenclature and an estimation of the abundance of IDRs and the prevalence of different subtypes (Necci *et al.*, 2016 ▸, 2018 ▸) have limited the integration of this knowledge within core data resources (CDRs) such as UniProtKB (The UniProt Consortium, 2021 ▸), Pfam (Mistry *et al.*, 2021 ▸), PDBe (PDBe-KB Consortium, 2020 ▸) and InterPro (Blum *et al.*, 2021 ▸). Only recently have highly confident disorder predictions such as those provided by the *MobiDB-lite* software (Necci, Piovesan, Clementel *et al.*, 2021 ▸) been integrated and made available in CDRs. However, high-quality IDR annotations from specialized databases remain significantly underrepresented and poorly cross-referenced.

**Figure 1:**
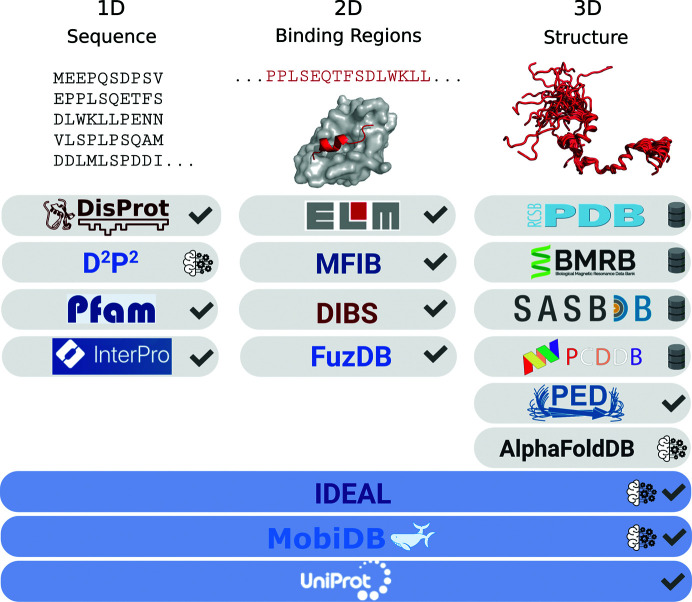
Overview of IDP/IDR data and the respective databases. The databases are organized according to the type of IDP/IDR data stored: sequence, binding regions and structural data. In the top part, examples are shown for each category using part of the N-terminal region of the human p53 protein (UniProtID p04637), its MDM2-binding short linear motif (ELM accession ELME000184 and PDB entry 1ycr, chain *B*, red) and the corresponding structural ensemble (PED ID PED00037e000). Curated databases are indicated with a check mark, deposition databases with a database icon and databases with predicted data with a machine-learning icon. Databases aggregating data from different sources have a light blue background. Created with *BioRender* (https://biorender.com/).

In this review, we present a comprehensive overview of the available IDR resources, highlighting differences related to their types, scopes, availability and sustainability. We describe (i) manually curated IDR databases, (ii) predicted IDR databases, (iii) deposition databases and (iv) liquid–liquid phase-separation databases. A comparative table describing the database content and coverage is provided for each category of IDR resources, as well as a schematic figure showing the databases organized according to the three different dimensions of IDP/IDR data (Fig. 1 ▸). We have also tried to highlight the trends in the development of these resources and the effort that has been made so far in connecting experimental results with IDRs.

## 2. Manually curated IDR databases

Manually curated resources are fundamental for IDRs, as experimental interpretation is challenging and a standard is lacking. In this section, we group all databases specific to manually curated IDR annotations (see Table 1 ▸). While having differences in scope, these generally focus on structural state attributes, binding properties and functions of IDRs.

DisProt (Hatos *et al.*, 2020 ▸) provides manually curated annotations of IDRs/IDPs. DisProt relies on both professional and community biocurators that focus on providing accurate, up-to-date and comprehensive annotations of intrinsically disordered proteins on a six-month release schedule. DisProt is cross-linked from several core databases such as UniProtKB. DisProt implements a comprehensive curation model including annotation of the structural (disordered) state, structural transitions, interaction partners and functions associated with IDRs. Each of these aspects is further expanded for finer classification. Functions annotated in DisProt are specific to IDPs and describe their involvement in the formation of biological condensates, in complex assembly or in localization and their ability to act as entropic chains. DisProt curators capture a broad range of different experimental techniques from different sources (articles) in order to provide orthogonal pieces of evidence, therefore increasing the reliability of its annotations.

IDEAL (Fukuchi *et al.*, 2014 ▸) is a database with manually curated annotations covering both structural and binding evidence for IDRs. IDEAL collects experimental data for protein intrinsic disorder mostly from missing residues observed in X-ray experiments and from NMR data. Experimental evidence is manually verified in order to minimize false positives and experimental artifacts. The database compiles valuable information about protein-interaction networks involving IDRs/IDPs, folding-upon-binding regions (described as protean segments) and post-translational modification sites. Additionally, IDEAL contains a prediction section showing domain and order–disorder predictions.

FuzDB annotates a subtype of IDRs called fuzzy regions with a role in the formation and function of protein complexes or higher-order assemblies (Miskei *et al.*, 2017 ▸). There are two main subtypes of fuzzy regions. The first includes static polymorphisms, where alternative conformations of the same interacting elements are stabilized within the assembly. The second subtype are dynamic binding regions that retain conformational freedom within the assembly upon interaction with a partner. For each record, FuzDB provides a description of the complex and its biological role along with literature references to experimental evidence about the interaction.

DIBS (Schad *et al.*, 2018 ▸) and MFIB (Fichó *et al.*, 2017 ▸) collect folding-upon-binding examples and consider PDB structures as a source of evidence for binding. DIBS and MFIB are complementary to each other. DIBS contains IDRs bound to globular protein partners and MFIB contains protein complexes entirely formed by IDPs. Their added value is the evidence for IDP-mediated interactions and the underlying mechanisms of their binding mode to ordered and unstructured partners, respectively. Both resources were last updated in 2017. A few examples of static polymorphisms, as defined by FuzDB, are also available in DIBS and MFIB, but they are not explicitly highlighted as such. All of the abovementioned databases are specifically focused on IDRs. In the following, we describe those databases that are indirectly or marginally related to disorder.

The eukaryotic linear motif (ELM) database is a repository of manually curated short linear motifs (SLiMs; Kumar *et al.*, 2020 ▸). SLiMs are interaction sites composed of short stretches of adjacent amino acids found within IDRs. In comparison with IDR binding regions available in other databases, these are more compact, shorter and are always associated with a specific function. In ELM, there are ∼300 different functions (‘classes’) associated with more than 3500 records (‘instances’). SLiMs have well defined functional roles, being conserved in different organisms by convergent evolution. Most of them exhibit a poorly conserved pattern, retaining only a few fixed positions. This characteristic, as well as their short lengths, makes their automatic detection in protein sequences difficult.

Pfam (Mistry *et al.*, 2021 ▸) is the main resource for protein families and domains. It provides protein annotations via hidden Markov models (HMMs) representing protein domains, which can be used to analyze entire proteomes efficiently. Each Pfam model is built on top of a manually curated seed alignment containing the representative sequences of the family and enriched with relevant biological information from the literature. Since release 34, Pfam flags protein families containing a high fraction of predicted disordered residues in their seed alignments as disordered.

Another source of curated IDR annotations is UniProtKB (The UniProt Consortium, 2021 ▸). IDR annotations are reported under the ‘Region’ section of the ‘Family and Domains’ block in the web page and correspond to the feature (‘FT’) section in the text format. Some IDR annotations are manually curated, while others are automatically transferred based on sequence similarity or by the *UniRule* algorithm (MacDougall *et al.*, 2020 ▸). The limited number of manually curated IDR annotations in UniProtKB is compensated by cross-references to DisProt (Hatos *et al.*, 2020 ▸), ELM (Kumar *et al.*, 2020 ▸) and MobiDB (Piovesan *et al.*, 2021 ▸). *MobiDB-lite* disorder predictions (Necci, Piovesan, Clementel *et al.*, 2021 ▸) were also recently added to UniProtKB as sequence features.

Of the manually curated IDR databases, DisProt is the most comprehensive, including both flexible linkers and multivalent or fuzzy interacting IDRs. DisProt annotations are not limited to disordered fragments, but include examples of fully dis­ordered proteins studied by circular dichroism, small-angle X-ray scattering (SAXS) or NMR chemical shift experiments (Felli & Pierattelli, 2015 ▸) which cannot be captured by crystallography or electron cryomicroscopy. DisProt is considered the gold standard for IDR annotation thanks to the high diversity and redundancy of IDR evidence. It is regularly used by experimental biologists to build hypotheses and by software developers to train and benchmark disorder predictors (Necci, Piovesan, CAID Preditors *et al.*, 2021 ▸). In addition, DisProt defines short and structurally linear binding interfaces in IDRs as linear interacting peptides (LIPs; Monzon *et al.*, 2021 ▸). These regions are crucial for IDRs to perform their function. The LIP definition used in DisProt acts as an umbrella term for SLiMs, intrinsically disordered binding regions, molecular recognition features, folding-upon-binding regions and fuzzy interactions.

## 3. Predicted IDR databases

IDRs can be predicted from the sequence by evaluating the local amino-acid composition. Higher accuracy can however be achieved by machine-learning or consensus methods and exploiting evolutionary information, as recently shown in the Critical Assessment of protein Intrinsic Disorder (CAID) experiment (Necci, Piovesan, CAID Preditors *et al.*, 2021 ▸). Prediction methods are made available as web servers or standalone packages, but obtaining the results can be problematic due to computational cost or to complications in installing and executing the software. IDR prediction databases providing precalculated results are a convenient solution to easily access disorder annotations and explore large data sets, for example for comparative analyses at the genome level.

MobiDB is the major knowledge base for IDRs and related annotations. It combines different types of annotations in a data-quality pyramid, providing a trade-off between quality and coverage. At the top of the pyramid are the relatively few manually curated IDR annotations from the databases listed in the previous section. These annotations are expanded to orthologous IDR sequences, for example p53 rat annotated from p53 human in DisProt. Indirect IDR annotations derived from the Protein Data Bank (PDB), such as missing residues in X-ray structures, are in the middle of the MobiDB data pyramid. While extracted from experimental structures, these annotations are not manually validated as in curated databases. *Mobi* (Piovesan & Tosatto, 2018 ▸) is used to infer disorder from the evaluation of missing and mobile residues. *FLIPPER* (Monzon *et al.*, 2021 ▸) is used to detect binding IDR annotations, which in MobiDB are called linear interacting peptides (LIPs).

IDR predictions provide the greatest coverage of protein sequences, covering all of UniProtKB, at the expense of more limited quality and form the bottom of the data pyramid in MobiDB. Eight different IDR predictors are computed for each available protein sequence and combined in *MobiDB-lite*, a meta predictor trained to favor precision over recall (Necci, Piovesan, Clementel *et al.*, 2021 ▸). *MobiDB-lite* predicts longer contiguous IDR regions which can be thought of as the opposite of a globular domain, recognizing seven different flavors of disorder from their sequence composition. *MobiDB-lite* predictions are also available in core databases such as InterPro (Blum *et al.*, 2021 ▸), UniProtKB (The UniProt Consortium, 2021 ▸) and PDBe (PDBe-KB Consortium, 2020 ▸). MobiDB also includes binding IDR predictions as provided by *ANCHOR* (Dosztányi *et al.*, 2009 ▸) and 15 other different methods for all of UniProtKB: ∼180 million sequences. MobiDB provides several types of consensus annotations to summarize the huge amount of information that it contains, such as data from the same source (for example multiple PDB entries for the same protein), of different provenance (for example predictions), derived data and manually curated evidence. MobiDB provides an API for programmatic access and extensive documentation about its content.

The InterPro database (Blum *et al.*, 2021 ▸) is a key resource providing protein-sequence classification into families, and recognizes conserved sites and key functional domains. The InterPro Consortium is integrated by many databases, which provide different signatures, into a single searchable resource. Across the integrated signatures, some of those from the Pfam database are flagged as disordered (see Tables 1 ▸ and 2 ▸). InterPro includes IDRs from *MobiDB-lite* (Necci, Piovesan, Clementel *et al.*, 2021 ▸) and is in sync with MobiDB. AlphaFoldDB (Tunyasuvunakool *et al.*, 2021 ▸) is an open-access resource of predicted protein structures released very recently that provides predictions for the entire human proteome and a few other model organisms. The prediction of structure is only partially complementary to disorder and can be used to infer IDRs by focusing on low-confidence predictions. Benchmarked with the CAID data (Necci, Piovesan, CAID Predictors *et al.*, 2021 ▸), *AlphaFold* has been recently shown to be competitive with state-of-the-art methods (Tunyasuvunakool *et al.*, 2021 ▸). Finally, the D^2^P^2^ database (Oates *et al.*, 2013 ▸) is conceptually very similar to MobiDB and includes IDR predictions. However, D^2^P^2^ covers a fraction of the MobiDB sequences, was last updated in 2015 and is no longer maintained.

## 4. Deposition databases

Deposition (or primary) databases store raw experimental data. While PDB structures provide direct evidence about stable conformations, they are also an invaluable source of information for IDR annotation. IDRs show little to no secondary structure in solution, ranging from molten globules to random coils (van der Lee *et al.*, 2014 ▸). Missing electron density in X-ray experiments corresponds to regions trapped in different conformations within the crystal lattice (Monzon *et al.*, 2020 ▸). A lack of dispersion of proton resonances and signal overlap in NMR spectra correspond to intramolecular motions that cause slower relaxation rates and allow the acquisition of spectra with narrow lines (Kachala *et al.*, 2015 ▸). Secondary chemical shifts, small-angle scattering and a number of other experimental techniques can provide indirect evidence of IDRs. Intrinsic disorder can also be derived from circular-dichroism data by predicting the secondary-structure content, although it is not possible to unambiguously assign the position of the IDRs as the spectra are an average of all contributing secondary-structural elements (Micsonai *et al.*, 2015 ▸).

The principal deposition databases for structural data are reported in Table 3 ▸. All databases are active but have a different coverage of published data. As every resolved structure is deposited in the PDB, knowledge about the coordinates of well structured proteins in the PDB records is complete. This is not the case for other types of experiments. While the Small Angle Scattering Biological Data Bank (SASBDB; Kikhney *et al.*, 2020 ▸), Biological Magnetic Resonance Bank (BMRB; Romero *et al.*, 2020 ▸) and Protein Circular Dichroism Data Bank (PCDDB; Whitmore *et al.*, 2017 ▸) guarantee to store primary data published in specialized journals, a large fraction of primary data published elsewhere nevertheless fails to be deposited. IDR annotation is manually curated for SASBDB but is not reported in the PDB, BMRB and PCDDB.

The Protein Ensemble Database (PED; Lazar *et al.*, 2021 ▸) is focused on protein ensembles representing conformation hetero­geneity and dynamic behavior of IDRs. PED contains integrative modeling experiments in which computational methods are employed to generate conformational ensembles starting from experimental constraints, mainly derived from NMR, SAXS and FRET. The ensembles are then processed, validated and associated with manually curated structural metadata to highlight IDRs and other structural properties. The latest version of PED implements a new submission process allowing users to submit their own ensembles through a web interface that implements an automated validation pipeline of the deposited ensemble. PED further provides qualitative and quantitative information about the ensembles, such as radius of gyration, secondary-structure populations, solvent accessibility and experimental conditions.

PDB-Dev is related to PED but is focused on structured proteins (Vallat *et al.*, 2018 ▸). PDB-Dev is a PDB project collecting structural models obtained through hybrid modeling: structural modeling based on a combination of experimental and computational techniques. It aims to establish mechanisms for processing and integrating hybrid models in the PDB. Both PED and PDB-Dev rely on integrative or hybrid modeling. Where PED is focused on the conformational heterogeneity in the IDR ensembles, PDB-Dev focuses on structured proteins which require integrative techniques that are not yet standardized.

In conclusion, deposition databases represent an invaluable, but only partially exploited, source of IDR information. On one hand, there is the problem that a large fraction of primary data on experimentally derived IDRs is not deposited, with the laudable exception of PDB structures. On the other hand, IDRs annotations from deposited CD and chemical shift data data are neither manually interpreted nor processed by any reliable pipeline. For example, secondary chemical shifts deposited in the BMRB can be used to infer secondary-structure propensities and, indirectly, IDRs (Sormanni *et al.*, 2017 ▸). A number of tools are available to extract this information by comparing chemical shift values with a reference. Given that defining the reference is problematic and the outputs of different tools diverge significantly, a database of precalculated IDRs from chemical shifts is not available. IDR knowledge from CD and chemical shift data is partially captured by manually curated databases, especially DisProt, that interpret experimental results reporting IDR positions and cross-references to the corresponding entries of the PCDDB and the BRMB. However, manual interpretation is difficult, often disputable, time-consuming and does not scale with data growth.

## 5. Liquid–liquid phase separation (LLPS) databases

The important role of IDPs/IDRs in mediating liquid–liquid phase separation (LLPS) and contributing to the formation of membraneless organelles has recently been established (Borcherds *et al.*, 2021 ▸) and a number of specialized databases have been deployed (see Table 4 ▸). These databases are wider in focus and scope, capturing different aspects of LLPS processes. All contain manually curated data and include additional information such as protein disorder, low complexity, experimental details, post-translational modifications, phase diagrams and subcellular locations, among others (Orti *et al.*, 2021 ▸). LLPS-associated proteins are classified based on their role in condensate formation as driver/scaffold, regulator and client. Drivers are proteins that can phase separate without the need for other cofactors. Regulators can switch the phase separation on or off (for example post-translational modification enzymes). Clients can partition into the organelle but do not influence its formation.

PhaSepDB (You *et al.*, 2020 ▸) aims to collect proteins that are found in membraneless organelles (MLOs) and organizes its entries based on the corresponding MLO location. All entries can be classified into three groups according to the quality of annotation: (i) reviewed (verified by PhaSepDB curators), (ii) UniProtKB reviewed (pulled from UniProtKB) and (iii) high-throughput (identified by high-throughput experiments). MloDisDB (Hou *et al.*, 2021 ▸) is a manually curated database developed by the same research group as PhaSepDB, but focusing on the association between MLOs and diseases.

PhaSePro (Mészáros *et al.*, 2020 ▸) is a resource of LLPS drivers. Each PhaSePro entry is manually curated and mapped to UniProtKB sequences. The most notable annotations stored in PhaSePro are the sequence boundaries of the driver region, molecular interactors of the protein, determinants of phase separation (for example environmental conditions), regulatory mechanisms (for example post-translational modifications) and mutations affecting the LLPS process.

LLPSDB (Li *et al.*, 2020 ▸) is a dedicated collection of *in vitro* LLPS experiments. The database includes the outcome and parameters of more than a thousand experiments. LLPSDB reports information about the protein construct and proteoform, which is particularly relevant in the case of LLPS measurements, which are often carried out under nonphysiological conditions. LLPSDB however lacks information concerning the *in vivo* biological context and the relevance of the stored proteins on the associated MLOs.

DrLLPS (Ning *et al.*, 2020 ▸) is a comprehensive resource of LLPS proteins from nine model organisms that collects and integrates information on different aspects, including IDPs, domain annotations, post-translational modifications, cancer mutations and molecular interactions, from over a hundred public resources. Proteins are classified by their role in the condensate (driver, regulator and client) and also based on their localization. More than 40 different condensates are reported (*in vitro* droplet, nucleus, cytoplasm, germ cell *etc.*).

Despite the various databases appearing to differ significantly in terms of the number of curated proteins, the number of driver proteins is almost the same and below 400. MobiDB integrates LLPS drivers from PhaSePro data, as it is fully manually curated and reports the regions responsible for the phase transition.

## 6. Future directions

We have provided an overview of the currently available IDP databases. These have matured considerably over the last decade both in number and in the depth of annotation, with a few clear trends emerging. As the width of phenomena encompassed by IDPs becomes clearer, specialized databases been proposed to capture the subtler differences. Linking IDRs with function has been a popular concept, epitomized by resources such as FuzDB, DIBS and ELM. Knowledge of proteins involved in phase separation has likewise produced a set of databases describing this phenomenon. This proliferation of databases is counterbalanced by the growth of some key resources, namely DisProt and MobiDB, which try to cover as much ground as possible for IDRs. DisProt provides high-quality curated data which can help us better understand the biophysical principles underpinning IDRs. It also provides the most comprehensive curation model, ranging from the experimental detection method to molecular function. DisProt is therefore currently seen as the gold standard in the field.

MobiDB, on the other hand, aims to combine as many different sources of IDR information as possible. Its unique data-quality pyramid allows it to aggregate different sources, forming a more complete picture, while increasing confidence in the annotations. The latter has not gone unnoticed, as several core databases, such as UniProtKB, InterPro and PDBe, have begun to integrate previously missing IDR information through *MobiDB-lite*. IDPs have finally started to become mainstream in the resources available to experimental biologists.

One area where more integration is still needed is connecting experimental results with IDRs. The PDB allows the automated large-scale extraction of useful proxies for IDRs, such as missing residues in X-ray structures and mobile regions in NMR ensembles, but is by definition biased towards structures. Integration of evidence for IDRs from other techniques such as SAXS, CD, FRET or NMR chemical shifts is still patchy at best. Since these are better able to describe IDR behavior, there is a need for better data interoperability and integration. The PED database offers an attempt to describe the ensemble nature of IDRs based on structural constraints from various experiments. However, more work is needed to fully describe the dynamic nature of IDRs. The IDPcentral consortium aims to fill this gap by connecting available IDR-related databases through a unified query interface. The IDPcentral registry (https://idpcentral.org/) will bring together the stakeholders in the intrinsic disorder field and aggregate data from core resources on IDPs, acting as a hub for users to access IDP-related predictions and high-quality manually curated annotations. As the available IDP databases improve, the next decade will bring a more quantitative understanding of the various IDP phenomena.

## Figures and Tables

**Table 1:** Manually curated intrinsic disorder databases The name and URL are provided for each database. Creation date corresponds to the year of the first publication describing the resource. The numbers of proteins with intrinsically disordered regions (IDRs) and linear interacting peptides (LIPs) are reported based on the database websites. Notice that some databases provide only IDRs or LIPs. Data were collected in October 2021.

			IDRs		LIPs	
Name	URL	Creation date	Proteins	Content (%)	Proteins	Content (%)
Pfam	http://pfam.xfam.org/	1997	39†	>80	—	—
UniProtKB	https://www.uniprot.org/	2002‡	475‡	13.8	—	—
ELM	http://elm.eu.org/	2003	—	—	3542	1.4
DisProt	https://disprot.org/	2005	1746	20.5	729	19.3
IDEAL	https://www.ideal-db.org/	2012	995	10.3	317	8.9
FuzDB	https://fuzdb.org/	2016	110§	16.6§	—	—
MFIB	http://mfib.enzim.ttk.mta.hu/	2017	—	—	205	24.7
DIBS	http://dibs.enzim.ttk.mta.hu/	2017	—	—	772	4.1

† Intrinsically disordered domain families available in Pfam release 34.

‡ UniProtKB proteins are those with at least one manually curated IDR. The release year indicated is when the UniProtKB consortium (Swiss-Prot, TrEMBL and PIR) was launched; however, the release year of Swiss-Prot is 1986.

§ FuzDB annotates IDRs that form protein complexes retaining conformational heterogeneity.

**Table 2:** Intrinsic disorder prediction databases Columns are the same as in Table 1 ▸. The ‘Proteins’ column indicates the total number of database proteins, while the ‘Annotated’ columns indicate proteins with at least one IDR or LIP. ‘MobiDB curated’ includes data from the combined DisProt, IDEAL, FuzDB, ELM, UniProtKB, DIBS and MFIB databases. ‘MobiDB derived’ are missing residues in PDB structures. ‘MobiDB predicted’ lists IDRs predicted by *MobiDB-lite* and LIPs predicted by *ANCHOR*. IDR and LIP content are the fraction of annotated residues in proteins with at least one annotated region (MobiDB statistics release 2020_09). D^2^P^2^ provides only IDR annotations (as shown on the website) and statistics at the residue level are not available (NA). InterPro proteins are those matching with disordered Pfam domains. Disorder content is calculated based on the residues covered by Pfam models flagged as intrinsically disordered. Notice that AlphaFoldDB (queried on 26th July, 2021) is growing daily until it covers all UniRef90 proteins. Data were collected in October 2021.

				IDRs		LIPs	
Name	URL	Creation date	Proteins	Annotated	Content (%)	Annotated	Content (%)
MobiDB curated	https://mobidb.org/	2012	NA	2074	16.7	2871	5.8
MobiDB derived	https://mobidb.org/	2012	NA	35136	6.0	8979	5.8
MobiDB predicted	https://mobidb.org/	2012	189525031	187222768	12.1	111772244	10.5
*MobiDB-lite*	https://mobidb.org/	2012	189525031	38542336	17.1	—	—
D^2^P^2^	https://d2p2.pro/	2014	10429761	NA	NA	—	—
InterPro	http://www.ebi.ac.uk/interpro/	2001	219740214	233001	26.2	—	—
AlphaFoldDB	https://alphafold.ebi.ac.uk/	2021	362094	NA	NA	—	—

**Table 3:** Deposition databases Deposition databases containing primary data are listed by name and URL. Creation date corresponds to the year of the first publication describing the resource, and all are actively maintained. The number of records correspond to different depositions of a particular type of data (X-ray, NMR, SAXS, CD *etc.*). Notice that these databases are redundant, *i.e.* the same data can be deposited more than once. Manually curated IDR annotations are provided by SASBDB and PED. Data were collected in October 2021.

Name	URL	Creation date	Records	Type of data
PDB	http://www.wwpdb.org/	1971	176247	X-ray, NMR, cryo-EM
BMRB	https://bmrb.io/	1989	14254	NMR chemical shifts
PCDDB	https://pcddb.cryst.bbk.ac.uk/	2006	697	Circular dichroism
SASBDB	https://www.sasbdb.org/	2014	1942	Small-angle scattering
PED	https://proteinensemble.org/	2014	152	Integrative modeling (disordered ensembles)
PDB-Dev	https://pdb-dev.wwpdb.org/	2018	58	Integrative modeling (structured proteins)

**Table 4:** LLPS databases The name and URL are provided for manually curated LLPS databases. Creation date corresponds to the year of the first publication describing the resource. All databases were developed in the last two years and are currently being maintained. The type of data represented by the total number of records is specified as each database has a different content. Data were collected in October 2021.

Name	URL	Creation date	Records	Type of data
PhaSepDB	http://db.phasep.pro/	2019	2957	MLO localization/association
MloDisDB	http://mlodis.phasep.pro/	2021	771	MLO localization/association and diseases
PhaSePro	https://phasepro.elte.hu/	2019	121	Drivers/scaffolds
LLPSDB	http://bio-comp.org.cn/llpsdb/home.html	2019	1175	Experiments
DrLLPS	http://llps.biocuckoo.cn/	2019	9285	Clients, regulators, drivers/scaffolds
